# Main challenges of the detection in the context of global health security: systematic review of Joint External Evaluation (JEE) reports

**DOI:** 10.11604/pamj.2022.42.243.26563

**Published:** 2022-07-29

**Authors:** Vincent Dossou Sodjinou, Paul Ahoumènou Ayelo, Alfred Douba, Dona Edgard-Marius Ouendo

**Affiliations:** 1 University of Abomey-Calavi, Regional Institute of Public Health of Ouidah, Ouidah, Benin,; 2 University of Abomey-Calavi, Medical Sciences Faculty of Cotonou, Cotonou, Benin,; 3 Félix Houphouët-Boigny University of Cocody, Cocody, Abidjan

**Keywords:** Joint External Evaluation, challenges, laboratory, surveillance, reporting, workforce development, WHO regions

## Abstract

**Introduction:**

since 2016, Joint External Evaluation (JEE) missions have been organized in various countries. This systematic review of the JEE reports is intended to identify the main challenges (MC) of detection in WHO regions.

**Methods:**

we accessed JEE reports on the WHO website. Challenge was defined as a variable of the indicators of detection where there was a need of improvement. MC was a challenge common to at least one-third of countries in each region and globally. For consistency, we assessed challenges reported under “Areas which need strengthening/challenges” in reports.

**Results:**

we analyzed 96 JEE reports. African Region (91.7%), Eastern Mediterranean Region (80.9%) and South East Asia Region (72.7%) had the highest rates of JEE completion. The MC were 24 in European Region, 26 in Mediterranean Region, 30 in Western Pacific Region, 33 in South East Asia Region and 34 in African Region. 24 MCs were identified at global level. National laboratory system and Real time surveillance had the highest number of MC. Eleven MCs were common to all WHO regions and global level. These include insufficient capacity for core test confirmation, insufficient specimen referral system, weak quality management system, issues in laboratories licensing and accreditation, weak data management, weak electronic reporting system, absence /weak mechanism of information exchange between International Health Regulation and animal health focal points, insufficient health professional specialists, the need of workforce strategy, the need of field epidemiology and insufficient workforce retention capacity.

**Conclusion:**

the MCs identified should be addressed through a global approach.

## Introduction

The Global Health Security (GHS) has been a matter of high interest since the middle of the 20^th^ century. This interest has increased since the largest Ebola virus disease outbreak occurred in West Africa from 2014 to 2016 [1]. To prevent or control public health treats, legal instrument such as international health regulation (IHR) 2005 was developed. The IHR 2005 was adopted in May 2005 by the fifty-eighth World Health Assembly as an effective mechanism to improve health security [2]. One of the most important provisions in the IHR was the obligation for all States Parties to establish core capacities to detect, assess, notify and report events, and to respond to public health risks and emergencies. The initial target date for establishment of these capacities was June 2012. At that time, 118 States Parties requested and were granted a two-year extension of the deadline up to June 2014 [3]. Unfortunately, the expected levels of capacities were not reached by numerous countries at the end of different extensions. In addition, the quality and the objectivity of the self-assessment were questionable [4]. To overcome these issues, new options were adopted by the Executive Board at its 136^th^ session. One of these options was the voluntary external evaluation of IHR [3]. Thus, a tool was developed to conduct Joint External Evaluation (JEE). The tool contains four domains/areas (Prevention, Detection, Response; and points of entry and Other IHR-related hazards) and is intended to assess country capacity to prevent, detect, and rapidly respond to public health threats [5].

Since 2016, JEE missions were organized in various countries. Reports of these missions are developed and displayed on the World Health Organization (WHO) website. A score ranging from 1 to 5 was granted to each technical area. The JEE tool includes 19 technical areas namely legislation, coordination, antimicrobial resistance, zoonosis, food safety, biosafety and biosecurity and immunization in prevention domain; national laboratory system (NLS), real time surveillance (RTS), reporting and workforce development in detection domain; emergency preparedness, emergency response operation, linking public health and security, medical countermeasures and personal deployment, risk communication in response; and point of entry, chemical events and radiation nuclear in point of entry and other IHR related hazards [5]. The countries JEE reports avail specific details on challenges per each technical area. But to our knowledge, there is so far no study conducted to assess the main challenges (MC) commonly reported per WHO region and globally. This systematic review is intended to fill this gap. The review focused on the detection domains and its four technical areas. This focus was mainly driven by two reasons. Firstly, due to time and resources constraints, it was not possible to work on all the domains at the same time. Secondly, the detection domain plays a core role in the GHS and is like an entry point for the GHS. If detection works well, the health system will have scientific evidences on the effectiveness of prevention interventions, will detect early any new events and will enable timely implementation of response interventions. That is why this study focused on detection. The objective of this work was then to identify the main challenges of the detection area in the WHO regions in a context of the Global Health Security Agenda.

## Methods

**Study design:** this is a descriptive systematic review of JEE reports.

**Setting:** locations included in the study were Africa, Eastern Mediterranean, Europe, Americas, South-East Asia and Western Pacific. JEE reports for missions conducted in these locations from 22 February 2016 to 12 July 2019 were included in the study.

**Participants:** the study population was the World Health Organization (WHO) regions. The targeted population were WHO regions where the voluntary JEE missions were organized. The non-probabilistic sampling method was used. All the WHO regions where JEE was conducted were included (exhaustive choice) and all the available reports were screened and analyzed.

**Figure 1:**
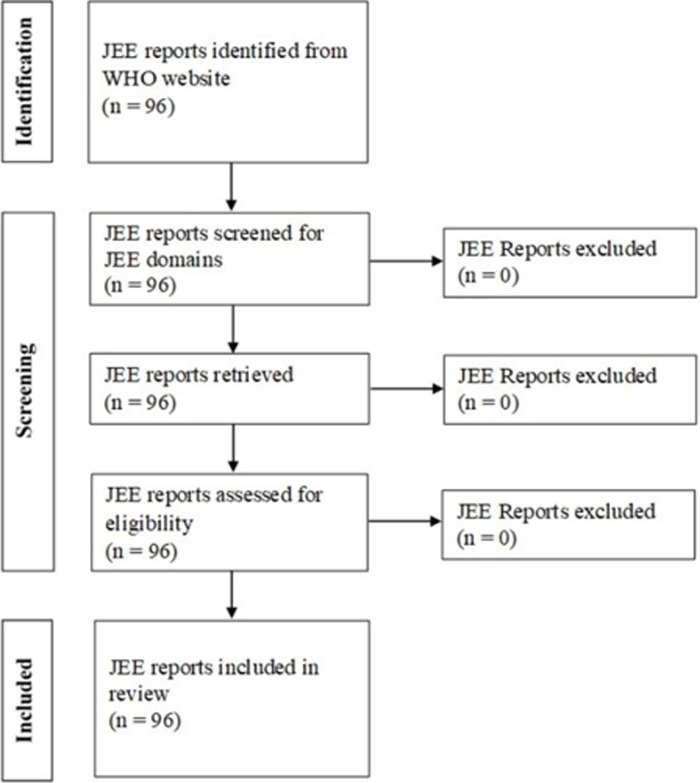
JEE reports selection process, May 2020

**Data sources:** to retrieve these reports, we accessed the WHO website and searched for “Joint External Evaluation reports”. We went on the WHO JEE reports storage home page. We clicked on each region and had access to JEE reports of countries who conducted the JEE and whose reports were published on WHO website by the time of our research in May 2020. JEE reports selection process is shown in Figure 1. We found JEE reports for African Region (AFRO) [6], Eastern Mediterranean Region (EMRO) [7], European Region (EURO) [8], WHO Region of the Americas (PAHO) [9], South-East Asia Region (SEARO) [10] and Western Pacific Region (WPRO) [11]. The JEE reports found were assessed using the Critical Appraisal Skills Program (CASP) qualitative research checklist [12]. The checklist has 10 questions, namely (i) was there a clear statement of the aims of the research? (ii) is a qualitative methodology appropriate? (iii) was the research design appropriate to address the aims of the research? (iv) was the recruitment strategy appropriate to the aims of the research? (v) was the data collected in a way that addressed the research issue? (vi) has the relationship between researcher and participants been adequately considered? (vii) have ethical issues been taken into consideration? (viii) was the data analysis sufficiently rigorous? (ix) is there a clear statement of findings? (x) how valuable is the research? A score of 1 was assigned to a positive answer to each question. Each report reaching 8 positive responses (80%) was included in the review. But overall, each JEE report was granted a score of 9 (90%) out of 10 (100%).

**Variables:** the outcome variable in this study was MCs identified in the JEE. Challenge was defined as a variable of indicators of actions packages of detection domain (NLS, RTS, Reporting, and Workforce development) where there was need of improvement. MC was defined as a challenge common to at least one-third of countries in each WHO region and globally.

**Bias:** the extraction of MCs from JEE reports was performed by two separate teams. To avoid or control MC selection bias, each team used the same definition of the MC. We developed an Excel database of all reported challenges in the JEE reports for detection domain per WHO region. The dataset included: WHO region, domain, action package, indicator, challenge and country. Document exploitation technique was used to identify challenges. There were differences in the JEE reports sections, with some reports having a section “challenges” for each package. To ensure consistencies, we selected challenges reported under the section “Areas which need strengthening/challenges” as this section was present in all the JEE reports. The domain sections of JEE reports were read in order to identify challenges. A content analysis of the formulation of the challenges was then performed to identify the MCs. Some JEE recommendations were not specific enough [13]. Efforts were then made to ensure specificity of the formulation of the challenges. Challenges reporting more than one idea were separated. Some challenges were discarded when the formulation was not clear. For example, the challenge “Resources for efficient NMC surveillance at provincial level need to be increased” formulated in South Africa report was discarded as it was not clear if resources mean “human resources” or “financial resources” or “material resources”. On other hands, some challenges were reclassified under the appropriate indicator when needed.

**Statistical methods:** the data was analyzed using Excel software. The proportion of challenges was computed per indicator and package by WHO regions and globally. As just two countries completed JEE missions in PAHO at the time of the study, this region was discarded for analysis of MCs.

## Results

The selection process (Figure 1) led to the identification of 96 JEE reports. The distribution of these reports per WHO regions is shown in Table 1. Globally, around half of the WHO Member States completed the JEE. A proportion of 46 % of the reports were from African region. The highest rates of JEE completion per region were found in AFRO, EMRO and SEARO. The cumulative number of challenges varies by WHO region. AFRO accounted for almost half of reported challenges (Table 1). The highest proportion of challenges were reported for NLS (32.1%) and RTS (31.2%) (Table 2). The number of challenges per indicator ranged from 173 (5.2 %) for syndromic surveillance to 362 challenges (10.8 %) for indicator and event-based surveillance. Indicators with highest number of challenges were (i) indicator and event-based surveillance (10.8 %), (ii) laboratory testing for detection of priority diseases (10.4 %), (iii) laboratory quality system (8.1%); and (iv) Inter-operable, interconnected, electronic real-time reporting system (8.1%) (Table 2).

**Table 1:** distribution of JEE reports and challenges by WHO region, May 2020

WHO Region	Number of Member States	Proportion of JEE completion	Number and proportion of JEE reports	Number and proportion of challenges
AFRO	48*	91.7%	44 (46.0%)	1635 (48.8%)
EMRO	21	80.9%	17 (17.7%)	585 (17.5%)
SEARO	11	72.7%	08 (08.3%)	483 (14.4%)
WPRO	27	40.7%	11 (11.4%)	342 (10.2%)
EURO	52	26.9%	14 (14.6%)	261 (07.8%)
PAHO	35	05.7%	02 (02.0%)	46 (01.4%)
Total	194	49.0%	96 (100.0%)	3352 (100.0%)

* including Zanzibar

**Table 2:** global distribution of challenges by packages and indicators for detection domain, May 2020

Packages	Indicators	Challenges	Proportion
D1. National laboratory system	D.1.1 Laboratory testing for detection of priority diseases	347	10.4%
	D.1.2 Specimen referral and transport system	226	06.7%
	D.1.3 Effective modern point-of-care and laboratory-based diagnostics	231	06.9%
	D.1.4 Laboratory quality system	272	08.1%
	**Subtotal D1**	**1076**	**32.1%**
D2. Real time surveillance	D.2.1 Indicator and event-based surveillance systems	362	10.8%
	D.2.2 Inter-operable, interconnected, electronic real-time reporting system	271	08.1%
	D.2.3 Analysis of surveillance data	241	07.2%
	D.2.4 Syndromic surveillance systems	173	05.2%
	**Subtotal D2**	**1047**	**31.2%**
D3. Reporting	D.3.1 System for efficient reporting to WHO, FAO and OIE	263	07.8%
	D.3.2 Reporting network and protocols in country	234	07.0%
	**Subtotal D3**	**497**	**14.8%**
D4. Workforce development	D.4.1 Human resources available to implement IHR core capacity requirements	263	07.8%
	D.4.2 FETP or other applied epidemiology training program in place	228	06.8%
	D.4.3 Workforce strategy	241	07.2%
	**Subtotal D4**	**732**	**21.9%**
	Total	3352	100.0%

**Figure 2:**
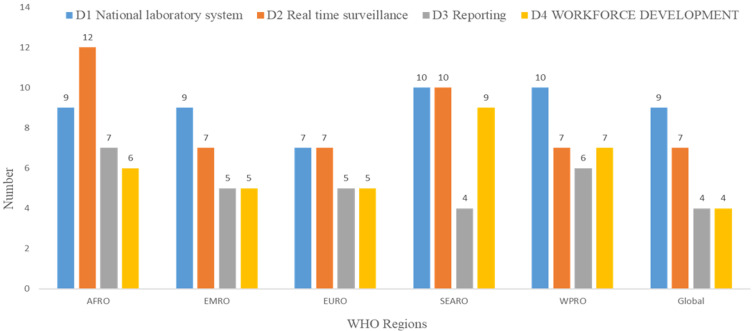
distribution of detection main challenges per packages by WHO regions and globally, May 2020

**Main challenges for national laboratory system:** a cumulative number of 45 MCs were identified for NLS. This included 9 MCs for AFRO, 9 for EMRO, 7 for EURO, 10 for SEARO and 10 for WPRO. At global level, 9 MCs were identified (Figure 2).

**Main challenges for real time surveillance:** a total of 43 MCs were identified for RTS. This included 12 MCs for AFRO, 7 for EMRO, 7 for EURO, 10 for SEARO and 7 for WPRO. At global level, 7 MCs were identified (Figure 2).

**Main challenges for Reporting:** a sum of 27 MCs were identified for reporting. This included 7 MCs for AFRO, 5 for EMRO, 5 for EURO, 4 for SEARO and 6 for WPRO. At global level, 4 MCs were identified (Figure 2).

**Main challenges for Workforce Development:** a cumulative number of 32 MCs were identified for workforce development. This included 6 MCs for AFRO, 5 for EMRO, 5 for EURO, 9 for SEARO and 7 for WPRO. At global level, 4 MCs were identified (Figure 2).

### Main challenges reported in all regions

Eleven (11) MCs were identified in all WHO regions and at global level (Table 3, Table 3(suite), Table 3(suite 1), Table 3(suite 2)). Four are reported in NLS namely (i) the insufficient capacity for core tests confirmation (equipment, structures), (ii) the insufficient functioning referral system in human and / or animal sector at all levels or from local level to reference laboratory, (iii) the weaknesses in external quality assurance (not mandatory for all laboratories, not or insufficiently implemented, some laboratories are not covered, some pathogens are not covered) and (iv) the issues about mandatory laboratories licensing and accreditation in the public and / or private sector. Two of the common MCs are reported in RTS namely (v) the weak data management (collation, validation, quality audits, completeness, promptness) at each level in human and / or animal sector and (vi) the absence or insufficient use of electronic reporting systems for notifiable diseases for human health and animal health. One common MC is identified in reporting action package namely (vii) the absence or insufficient mechanism ensuring that IHR NFP and OIE contact points exchange information when needed including on zoonotic diseases (no SOP, exchange not formalized, etc.). Four common MCs are identified in the workforce development action package. These are (viii) the insufficient number of health professional specialists with competencies in surveillance and epidemiology, laboratory and veterinary services, (ix) the need of basic, intermediate or advanced FELTP course and the need of more specialized epidemiological courses, (x) the need to develop, update and monitor health workforce strategy and human resource plan and (xi) insufficient incentives, strategies and efforts to maintain and retain the existing public health workforce.

**Table 3:** distribution of challenges per indicators and packages by WHO regions and globally, May 2020

Package	Indicator	Challenges	AFRO (N=44)	EMRO (N=17)	EURO (N=14)	SEARO (N=8)	WPRO (N=11)	Global (N=94)
D.3 Reporting	D.3.1 System for efficient reporting to WHO, FAO and OIE	No / insufficient mechanism ensuring that IHR NFP and OIE Contact Points exchange information when needed including zoonotic disease (no SOP, exchange not formalized, etc.)	31(70%)	9(53%)	7(50%)	5(63%)	4(36%)	56(60%)
		IHR NFP not or insufficiently trained on his specific role	20(45%)	4(24%)	5(38%)	4(50%)	4(36%)	36(38%)
		IHR NFP not operational	18(41%)	5(29%)	1(7%)	1(13%)	0(0%)	25(27%)
		Absence / insufficient formal electronic system for sharing information between the animal and human health sectors and with other relevant sectors.	15(34%)	2(12%)	2(14%)	1(13%)	0(0%)	20(21%)
		No or insufficient capacity to conduct risk assessments for public health events of chemical and radiation origin and events of unknown origin.	8(18%)	7(41%)	0(0%)	0(0%)	5(45%)	20(21%)
		Insufficient national capacity to identify and report PHEIC to WHO within 24 hours.	2(5%)	6(35%)	2(14%)	1(13%)	0(0%)	11(12%)
		Insufficient cross-sectoral coordination system for reporting to the IHR NFP	2(5%)	1(6%)	0(0%)	5(63%)	0(0%)	8(9%)
	D.3.2 Reporting network and protocols in country	Absence / need of protocols or procedures for reporting of public health event to WHO, OIE and FAO	30(68%)	4(24%)	2(14%)	2(25%)	3(27%)	41(44%)
		No legislation or other policies related to procedures and/or approvals for reporting on a potential PHEIC to the WHO, FAO, OIE	18(41%)	6(35%)	8(57%)	5(63%)	0(0%)	37(39%)
		Insufficient periodic simulation exercises involving all relevant stakeholders at all levels	15(34%)	1(6%)	5(36%)	0(0%)	4(36%)	25(27%)
		Lack of awareness of the decision instrument (Annex 2 of IHR) and its use among the non-health sector.	0(0%)	5(29%)	7(50%)	1(13%)	5(45%)	18(19%)

N represents the number of countries that completed JEE in each region. Blue cells indicate main challenges.

**Table 3(suite):** distribution of challenges per indicators and packages by WHO regions and globally, May 2020

Package	Indicator	Challenges	AFRO (N=44)	EMRO (N=17)	EURO (N=14)	SEARO (N=8)	WPRO (N=11)	Global (N=94)
D.2 Real time surveillance	D.2.1 Indicator and event-based surveillance	No or insufficient event-based surveillance (human health sector, animal sector, environment sector, insufficient geographical coverage, insufficient implementation, no list of priority event or case definition)	30(68%)	15(88%)	6(43%)	0(0%)	4(36%)	55(59%)
		Weak / insufficient community-based surveillance in all provinces	21(48%)	1(6%)	6(43%)	1(13%)	0(0%)	29(31%)
		Weak data management (collation, validation, quality audits, completeness, promptness) at each level in human and / or animal sector	20(45%)	7(41%)	7(50%)	3(38%)	4(36%)	41(44%)
		Low involvement of hospitals and / or private sector in surveillance	15(34%)	5(29%)	0(0%)	3(38%)	1(9%)	24(26%)
		Insufficient timeliness of reporting / complete and timely surveillance reports	9(20%)	1(6%)	4(29%)	5(63%)	0(0%)	19(20%)
	D.2.2 Interoperable, interconnected, electronic real-time reporting system	Need of training on surveillance (Health workers, community members, private sector, IDSR, maintenance, event-based surveillance)	21(48%)	4(24%)	5(36%)	3(38%)	4(36%)	37(39%)
		No or insufficient use of electronic reporting systems for notifiable diseases for human health and animal health	20(45%)	15(88%)	9(64%)	7(88%)	7(64%)	58(62%)
		Human surveillance system is not or is insufficiently interconnected and interoperable with animal and environment sectors surveillance	28(64%)	10(59%)	4(29%)	3(38%)	6(55%)	51(54%)
		Collaboration between the human and animal health sectors in the area of zoonotic diseases should be strengthened	15(34%)	5(29%)	6(43%)	2(25%)	5(45%)	33(35%)
		Weak internet connectivity in the health facilities and / or low availability of IT materials.	8(18%)	3(18%)	0(0%)	1(13%)	4(36%)	16(17%)
	D.2.3 Analysis of surveillance data	Insufficient capacity of surveillance officers on data analysis at the district level	21(48%)	10(59%)	4(29%)	5(63%)	4(36%)	44(47%)
		No mechanism in place to link epidemiological and laboratory data	15(34%)	7(41%)	0(0%)	2(25%)	2(18%)	26(28%)
		No centrally located mechanism for integrating data from clinical case reporting and data from clinical or reference microbiological laboratories	15(34%)	1(6%)	0(0%)	4(50%)	0(0%)	20(21%)
		No / insufficient analysis of surveillance data at district level	15(34%)	4(24%)	3(21%)	2(25%)	2(18%)	26(28%)
		Insufficient development of complete and timely report by each surveillance system (Publishing and disseminating surveillance reports or bulletins on a weekly basis)	7(16%)	4(24%)	0(0%)	3(38%)	0(0%)	14(15%)
	D.2.4 Syndromic surveillance	No or weak syndromic surveillance	12(27%)	5(29%)	5(36%)	3(38%)	2(18%)	27(29%)
		Reporting should be systematically shared with relevant sectors	16(36%)	4(24%)	1(7%)	2(25%)	0(0%)	23(24%)

N represents the number of countries that completed JEE in each region. Blue cells indicate main challenges.

**Table 3(suite 1):** distribution of challenges per indicators and packages by WHO regions and globally, May 2020

Package	Indicator	Challenges	AFRO (N=44)	EMRO (N=17)	EURO (N=14)	SEARO (N=8)	WPRO (N=11)	Global (N=94)
D.3 Reporting	D.3.1 System for efficient reporting to WHO, FAO and OIE	No / insufficient mechanism ensuring that IHR NFP and OIE Contact Points exchange information when needed including zoonotic disease (no SOP, exchange not formalized, etc.)	31(70%)	9(53%)	7(50%)	5(63%)	4(36%)	56(60%)
		IHR NFP not or insufficiently trained on his specific role	20(45%)	4(24%)	5(38%)	4(50%)	4(36%)	36(38%)
		IHR NFP not operational	18(41%)	5(29%)	1(7%)	1(13%)	0(0%)	25(27%)
		Absence / insufficient formal electronic system for sharing information between the animal and human health sectors and with other relevant sectors.	15(34%)	2(12%)	2(14%)	1(13%)	0(0%)	20(21%)
		No or insufficient capacity to conduct risk assessments for public health events of chemical and radiation origin and events of unknown origin.	8(18%)	7(41%)	0(0%)	0(0%)	5(45%)	20(21%)
		Insufficient national capacity to identify and report PHEIC to WHO within 24 hours.	2(5%)	6(35%)	2(14%)	1(13%)	0(0%)	11(12%)
		Insufficient cross-sectoral coordination system for reporting to the IHR NFP	2(5%)	1(6%)	0(0%)	5(63%)	0(0%)	8(9%)
	D.3.2 Reporting network and protocols in country	Absence / need of protocols or procedures for reporting of public health event to WHO, OIE and FAO	30(68%)	4(24%)	2(14%)	2(25%)	3(27%)	41(44%)
		No legislation or other policies related to procedures and/or approvals for reporting on a potential PHEIC to the WHO, FAO, OIE	18(41%)	6(35%)	8(57%)	5(63%)	0(0%)	37(39%)
		Insufficient periodic simulation exercises involving all relevant stakeholders at all levels	15(34%)	1(6%)	5(36%)	0(0%)	4(36%)	25(27%)
		Lack of awareness of the decision instrument (Annex 2 of IHR) and its use among the non-health sector.	0(0%)	5(29%)	7(50%)	1(13%)	5(45%)	18(19%)

N represents the number of countries that completed JEE in each region. Blue cells indicate main challenges.

**Table 3(suite 2):** distribution of challenges per indicators and packages by WHO regions and globally, May 2020

Package	Indicator	Challenges	AFRO (N=44)	EMRO (N=17)	EURO (N=14)	SEARO (N=8)	WPRO (N=11)	Global (N=94)
D.4 Workforce development	D.4.1 Human resources are available to implement IHR core capacity requirements	Insufficient health professional specialists with competencies in surveillance and epidemiology, Laboratory and veterinary services	31(70%)	11(65%)	6(43%)	4(50%)	7(64%)	59(63%)
		Unequal repartition of human resources in districts and local levels	15(34%)	5(29%)	2(14%)	3(38%)	4(36%)	29(31%)
		Public health professions are perceived as being less attractive than health care professions with level of remuneration is perceived as low	1(2%)	1(6%)	5(36%)	0(0%)	0(0%)	7(7%)
		Insufficient / no formalized coordination between the human and animal sectors on workforce development	0(0%)	2(12%)	0(0%)	3(38%)	0(0%)	5(5%)
		High turn-over of public health staff / HR	4(9%)	4(24%)	2(14%)	2(25%)	4(36%)	16(17%)
	D.4.2 Field epidemiology training program or other applied epidemiology training program in place	FETP course: No FETP- need of intermediate or advanced course - need of more specialized epidemiological course	26(59%)	11(65%)	8(57%)	4(50%)	6(55%)	55(59%)
		Low inclusion of nurses, animal sector staffs, lab and other in FELTP	11(25%)	5(29%)	3(21%)	3(38%)	2(18%)	24(26%)
		Participation in regional and international applied epidemiology activities should be enhanced (Cross border collaboration / international collaboration mechanism)	2(5%)	6(35%)	0(0%)	0(0%)	1(9%)	9(10%)
		Funding issues for FETP implementation	7(16%)	1(6%)	2(14%)	3(38%)	2(18%)	15(16%)
	D.4.3 Workforce strategy	Need to update or develop, monitor Health workforce strategy - HR plan	31(70%)	12(71%)	10(71%)	6(75%)	5(45%)	64(68%)
		Insufficient incentives, strategies and efforts to maintain and retain the existing public health workforce	23(52%)	8(47%)	8(57%)	6(75%)	5(45%)	50(53%)
		High attrition rate Insufficient incentive packages for staff posted to rural areas	15(34%)	2(12%)	4(29%)	1(13%)	1(9%)	23(24%)
		No clear career pathways or plan for public health workforce (epidemiologist, FETP)	9(20%)	4(24%)	4(29%)	6(75%)	4(36%)	27(29%)

N represents the number of countries that completed JEE in each region. Blue cells indicate main challenges.

## Discussion

This study aimed to identify MC of detection domain reported during JEE missions conducted in WHO regions. In summary, a total of 24 MCs were identified globally (Figure 2). Per region, the number of MC was 24 in EURO, 26 in EMRO, 30 in WPRO; 33 in SEARO and 34 in AFRO. Cumulatively, the leading packages were NLS (30.6%) and RTS (29.3%). Limitations of this study are intrinsic to the JEE process and / or reports. For example, the JEE process includes self-analysis by national teams followed by the external mission. It was reported that some national counterparts had inadequate understanding of the JEE process [14]; on the other hands, lessons learnt in Uganda from the process included the need to sufficiently orient and train subject matter experts [13]. Finally, the variability of the mission teams could have led to inconsistencies in the methods.

NLS appears as the first main detection challenging capacity in all WHO regions and globally. This capacity concentrates the highest cumulative number of MCs of detection across the world as defined by this study. This is in accordance with findings of other studies [15,16]. The situation of laboratory system seems to be the same in WHO regions. This can be surprising for EURO but the high number of countries that perform JEE exercises in this region were eastern European countries where health systems are less developed than in the remaining part of the region. In fact, the overall in-country laboratory capacity is relatively low across regions. Gaps are reported at national level as well as at local levels [17]. Capacities at local levels are very low with insufficient point of care capacities. This is in accordance with the variability of testing performance within administrative level reported in China [18]. Laboratory infrastructures are insufficient or ageing mainly in AFRO. Capacity for confirmation of emerging pathogens is relatively low and equipment are missing. This was evidenced by the delay reported in AFRO at the beginning of the covid-19 pandemic with just two countries able to confirm the covid-19 disease in February 2020 [19-21]. The absence of laboratory quality system in many countries poses additional problem about the quality of test performed [17]. Insufficient maintenance and calibration capacities are largely reported. Available capacities are concentrated at national level with insufficient point of care testing capacities. The situation is more concerning in animal, environmental and other sectors. Zoonotic disease capacities are very low compared to the capacity in human health area [3]. Countries seem to be more committed for human health and less efforts are deployed to improve the One Health approach. Another major factor is the insufficient workforce in laboratory area. Despite the fact that human resources are critical to strengthening laboratory systems [22], gaps in specialists are reported across the regions. During the national rapid assessment of laboratory capacity and systems in Sierra Leone in 2015, inadequate numbers of appropriately trained laboratorians were reported as well as the absence of a single laboratory worker in 30% of community health facilities [23,24]. This lack of resources and trained public health professionals poses a substantial roadblock. Regarding the core place and roles of laboratory system in event confirmation, the current status of laboratory capacity across regions is a huge threat for the global health security. There are large differences in laboratory capacity between WHO regions and countries. This situation causes delay in event confirmation in less developed countries, leading to the delay in the adequate response implementation. Developed countries and partners should support the less developed countries in building strong laboratory system.

A high number of MCs were also reported for RTS. AFRO and SEARO are the most challenging WHO regions. The insufficient implementation of event-based surveillance was largely reported in AFRO, EMRO, EURO and WPRO. In addition, existing event-based surveillance need to be extended geographically and need to cover more events, including environmental events. Knowing the importance of alerts for quick detection and reporting, this weak event-based surveillance will probably delay the detection of new events and the establishment of response interventions. The implementation of adequate measures to improve this area of surveillance will improve countries ability to contribute to the global health security [25-29]. Data management, analysis and use for decision-making is another area of improvement [29]. There is general lack of capacity for data analysis at district and local levels in all the WHO regions. Consequently, evidence-based decision-making is insufficiently performed and can lead to misuse of the scarce resources available. Training of workforce on data analysis and on surveillance is a key challenge across the regions. The health workers as well as the community members and the private sector staff need capacity building on integrated disease surveillance and response (IDSR) including event-based surveillance [26,27].

The issues about electronic surveillance are also important to be addressed. Real-time systems worked well in settings with good electronic and telecommunications infrastructure, while delays were common in settings with more limited infrastructure [30]. According to Holmgren *et al.*, the most prevalent barriers to electronic reporting were that public health agencies lacked the capacity to electronically receive data, interface-related issues (costs, complexity) and difficulty in extracting data from the electronic health record [31]. Access to internet and information technology equipment is challenging in AFRO and in other countries across the world. In 2017, the sub-Saharan Africa, southern and Central Asia had the lowest levels of internet penetration and wireless broadband infrastructure per capita, relative to other regions of the world [32]. Other barriers include the funding of the electronic system. In Sierra Leone, the total economic cost to roll out electronic IDSR (eIDSR) in the Western Area Rural district over a 14-week period was 64,342 United States Dollars (USD) with a per health facility cost of 1,021 USD. Equipment for eIDSR was the primary cost driver (45.5%) [33]. The largest part of this funding was provided by donors. This is in accordance with the heavy dependence on donors raised for the funding of many activities during JEE. Reporting is also a challenging capacity across WHO regions. The weaknesses in electronic surveillance are probably one of the factors explaining this situation, as well as the insufficient interoperability and interconnection among human and animal surveillance. The coordination among stakeholders is frequently missing. IHR focal point is frequently restricted to a single staff with no connection with relevant sectors. Available information exchange mechanism between IHR NFP and OIE contact person is not fully functioning. There is a need to improve national bridging workshops on the IHR (2005), and the OIE Performance of Veterinary Services Pathway [34] as well as training of IHR national focal points on their responsibilities [25,30].

The improvement of the workforce is one of the major enabling environments for the event detection. Workforce development was mostly challenging in SEARO and WPRO. AFRO appears in third position for this package. These are the results of efforts undertaken in this region to train on the IDSR, in field epidemiology program as well as in other related areas. The lack of needed specialists is largely reported across WHO regions. Reported human resources missing profiles include epidemiologists, biostatisticians, social scientist, occupational health, information technology specialist, biomedical technicians, maintenance officers, veterinarians and community nursing experts. This is probably linked with the absence of updated workforce strategy with insufficient training program on field epidemiology. On the other hand, the available workforce strategies are not implemented or monitored. Some of the careers that are highly important for IHR are not considered in workforce strategy. In developed countries, public health professions are less attractive, insufficiently valued and promoted. This led to insufficient critical mass of specialists in this core area for public health security. One major point on workforce is the need to improve the retention of specialists. There is urgent need to solve issues about insufficient incentives, strategies and efforts to maintain and retain the existing public health workforce in countries in WHO regions. Better career pathways for public health workforce will play a key role in this way.

Other enabling environment components need to be established or strengthened to enable improvement in detection domain. Legislations and standard operating procedures (SOP) for notification of potential public health event of international concern (PHEIC) to WHO as well as for other key activities such as samples collection, packaging and transport are still missing in some countries. National coordination bodies are not established or not functioning. This demonstrates that detection domain is linked with the other domains of global health security and IHR. Improvement in legislation and policies will positively impact detection capacities in WHO regions and countries. The level of specification about each MC differs across countries and regions. The MCs of detection identified should be deeply analyzed by countries with partners´ support during the development and implementation of the national actions plans for health security (NAPHS). Despite some missions had been organized since 2016, the findings remain valid in many countries. In fact, some countries (mostly in less developed countries) had not yet finalized their NAPHS. Just 13 validated and published NAPHS (including 7 for Afro Member States) were available on the WHO website at the time of this study [35,36]. In addition, funding issues are plaguing the implementation of the validated plans. This issue needs to be resolved. Africa region has the highest rate of JEE completion [14] followed by EMRO and SEARO. This enthusiastic commitment should be encouraged through adequate financial and technical support for the implementation of corrective actions. In fact, in the current context of travel celerity, any imbalance between WHO regions poses a threat to all WHO regions and countries. Strategic decision or resolutions should be taken by partners, donors and government to enforce Member States to allow a part of their national budget to the implementation of GHS major activities.

The aim of the study was achieved. The MCs of detection were identified per indicator, per region and globally. The study method was conducted in respect of the principles of systematic review. The potential inherent biases about the JEE missions don´t have huge impact on the validity of the results. However, the number of countries involved in the process for some regions is very low. This makes difficult to generalize the results for these regions. Although the objective was not to generalize the results, the MC identified could reflect the regional situation in AFRO, EMRO and SEARO as well as the situation at global level. The focus on the detection domain can also lead to the missing of information about other domains that can help in better understanding of the detection challenges.

## Conclusion

The systematic review of the JEE report enables the identification of MCs of the detection in WHO Regions. A total of 34 MCs were identified in AFRO including 9 for NLS, 12 for RTS, 7 for reporting and 6 for workforce development. A total of 26 MCs were identified in EMRO with 9 for NLS, 7 for RTS, 5 for reporting and 5 for workforce development. A total of 24 MCs were identified in EURO with 7 for NLS, 7 for RTS, 5 for reporting and 5 for workforce development. In SEARO, a total of 33 MCs were identified including 10 for NLS, 10 for RTS, 4 for reporting and 9 for workforce development. In WPRO, a total of 30 MCs were identified including 10 for NLS, 7 for RTS, 6 for reporting and 7 for workforce development. At global level, 24 MCs were identified including 9 for NLS, 7 for RTS, 4 for reporting and 4 for workforce development. Eleven (11) MCs were identified in all WHO regions and at global level. Four are reported in NLS namely (i) the insufficient capacity for core tests´ confirmation (equipment, structures), (ii) the insufficient functioning referral system in human and / or animal sector at all levels or from local level to reference laboratory, (iii) the weaknesses in external quality assurance (not mandatory for all laboratories, not or insufficiently implemented, some laboratories are not covered, some pathogens are not covered) and (iv) the issues about mandatory laboratories´ licensing and accreditation in the public and / or private sector. Two of the common MCs are reported in RTS namely (v) the weak data management (collation, validation, quality audits, completeness, promptness) at each level in human and / or animal sector and (vi) the absence or insufficient use of electronic reporting systems for notifiable diseases for human health and animal health. One common MC is identified in reporting action package namely (vii) the absence or insufficient mechanism ensuring that IHR NFP and OIE contact points exchange information when needed, including on zoonotic diseases (no SOP, exchange not formalized, etc.). Four common MCs are identified in workforce development action package. These are (viii) the insufficient number of health professional specialists with competencies in surveillance and epidemiology, laboratory and veterinary services, (ix) the need of basic, intermediate or advanced FELTP course and the need of more specialized epidemiological courses, (x) the need to develop, update and monitor health workforce strategy and human resource plan and (xi) insufficient incentives, strategies and efforts to maintain and retain the existing public health workforce. The study was intended to contribute to the improvement of the global health security. By focusing on the areas to be improved, the study is not denying huge efforts and improvements reported in the IHR capacities in the last decade. The MCs identified should be addressed through global approach to improve countries detection capacity in all regions, especially in Africa. It will also be useful to conduct similar research on the other domains of the global health security agenda.

### What is known about this topic

- Implementation of global health security faces challenges in each country;
- Identification of challenges guides decision making to improve global health security;
- Detection is a key element in the global health security since early detection of a public health emergency is crucial to avoid its extension across borders.

### What this study adds

- Identification of detection MC per each WHO regions and globally;
- National laboratory surveillance and real-time surveillance are the two fields with highest number of MC in each WHO regions and globally;
- African and South East Asia Regions are regions with highest number of MC related to national laboratory and real-time surveillance.

## References

[1] Heymann DL, Chen L, Takemi K, Fidler DP, Tappero JW, Thomas MJ (2015). Global health security: the wider lessons from the West African Ebola virus disease epidemic. The Lancet.

[2] World Health Organization (2008). International Health Regulations - 2^nd^ edition.

[3] World Health Organization (2015). Implementation of the International Health Regulations (2005): Responding to public health emergencies. 68 World Health Assembly report A68/22.

[4] Tsai FJ, Turbat B (2020). Is countries´ transparency associated with gaps between countries´ self and external evaluations for IHR core capacity?. Global Health.

[5] World Health Organization (2018). Joint External Evaluation tool - second edition.

[6] World Health Organization (2020). WHO African Region: JEE mission reports.

[7] World Health Organization (2020). WHO Eastern Mediterranean Region: JEE mission reports.

[8] World Health Organization (2020). WHO European Region: JEE mission reports.

[9] World Health Organization (2020). WHO Region of the Americas: JEE mission reports.

[10] World Health Organization (2020). WHO South-East Asia Region: JEE mission reports.

[11] World Health Organization (2020). WHO Western Pacific Region: JEE mission reports.

[12] Critical Appraisal Skills Programme CASP systematic review checklist.

[13] Kayiwa J, Kasule JN, Ario AR, Sendagire S, Homsy J, Lubwama B (2019). Conducting the Joint External Evaluation in Uganda: The process and lessons learned. Health Secur.

[14] Talisuna A, Yahaya AA, Rajatonirina SC, Stephen M, Oke A, Mpairwe A (2019). Joint external evaluation of the International Health Regulation 2005 capacities: current status and lessons learnt in the WHO African region. BMJ Global Health.

[15] Fossouo V, Mouiche M, Tiwoda C, Mba S, Wango R, Njayou A (2020). Assessing countries capacity for public health emergencies preparedness and response: the Joint External Evaluation process in Cameroon. Pan African Medical Journal.

[16] Borchert JN, Tappero JW, Downing R, Shoemaker T, Behumbiize P, Aceng J (2014). Rapidly building global health security capacity-Uganda demonstration project, 2013. MMWR.

[17] Hunsperger E, Juma B, Onyango C, Ochieng JB, Omballa V, Fields BS (2019). Building laboratory capacity to detect and characterize pathogens of public and global health security concern in Kenya. BMC Public Health.

[18] Liu B, Ma F, Rainey JJ, Liu X, Klena J, Liu X (2019). Capacity assessment of the health laboratory system in two resource-limited provinces in China. BMC Public Health.

[19] Ondoa P, Kebede Y, Loembe M, Bhiman J, Tessema S, Sow A (2020). COVID-19 testing in Africa: lessons learnt. Lancet Microbe.

[20] Lu R, Zhao X, Li J, Niu P, Yang B, Wu H (2020). Genomic characterisation and epidemiology of 2019 novel coronavirus: implications for virus origins and receptor binding. The Lancet.

[21] Zhu N, Zhang D, Wang W, Li X, Yang B, Song J (2020). A Novel coronavirus from patients with pneumonia in China, 2019. N Engl J Med.

[22] Mangal C, Maryogo L (2014). Leveraging the laboratory response network model for the global health security agenda. Biosecurity Bioterrorism Biodefense Strategy Practice and Science.

[23] Ministry of Health and Sanitation of Sierra Leone (2016). National rapid assessment of laboratory capacity and systems: approved report.

[24] Wurie I (2016). Sierra Leone laboratory systems-now and future. Afr J Lab Med.

[25] Keller M, Blench M, Tolentino H, Freifeld C, Mandl K, Mawudeku A (2009). Use of unstructured event-based reports for global infectious disease surveillance. Emerg Infect Dis.

[26] Ratnayake R, Crowe S, Jasperse J, Privette G, Stone E, Miller L (2016). Assessment of community event-based surveillance for Ebola virus disease, Sierra Leone, 2015. Emerg Infect Dis.

[27] Dagina R, Murhekar M, Rosewell A, Pavlin B, Event-based surveillance in Papua New Guinea: strengthening an International Health Regulations (2013). (2005) core capacity. Western Pac Surveill Response J.

[28] Hoen A, Keller M, Verma A, Buckeridge D, Brownstein J (2012). Electronic event-based surveillance for monitoring dengue, Latin America. Emerg Infect Dis.

[29] Clara A, Do TT, Dao ATP, Tran PD, Dang TQ, Tran QD (2018). Event-based surveillance at community and healthcare facilities, Vietnam, 2016-2017. Emerg Infect Dis.

[30] Suthar AB, Allen LG, Cifuentes S, Dye C, Nagata JM (2018). Lessons learnt from implementation of the International Health Regulations: a systematic review. Bull World Health Organ.

[31] Holmgren J, Apathy N, Adler J (2020). Barriers to hospital electronic public health reporting and implications for the COVID-19 pandemic. J Am Med Inform Assoc.

[32] Odusanya K, Adetutu M (2020). Exploring the determinants of internet usage in Nigeria: a micro-spatial approach. Responsible design implementation and use of information and communication technology - I. 3E edition.

[33] Sloan M, Gleason B, Squire J, Koroma F, Sogbeh S, Park M (2020). Cost analysis of health facility electronic integrated diseases surveillance and response in one district in Sierra Leone. Health Secur.

[34] World Health Organization (2019). Public health emergencies: preparedness and response. 72 World Health Assembly report A72/8.

[35] World Health Organization Strategic Partnership for International Health Regulation (2005) and Health Security (SPH): building stronger health systems for sustainable health Security.

[36] Mghamba J, Talisuna A, Suryantoro L, Saguti G, Muita M, Bakari M (2018). Developing a multisectoral National Action Plan for Health Security (NAPHS) to implement the International Health Regulations (IHR 2005) in Tanzania. BMJ Glob Health.

